# Toward Developing Techniques—Agnostic Machine Learning Classification Models for Forensically Relevant Glass Fragments

**DOI:** 10.1021/acs.jcim.2c01362

**Published:** 2022-12-13

**Authors:** Omer Kaspi, Osnat Israelsohn-Azulay, Zidon Yigal, Hila Rosengarten, Matea Krmpotić, Sabrina Gouasmia, Iva Bogdanović Radović, Pasi Jalkanen, Anna Liski, Kenichiro Mizohata, Jyrki Räisänen, Zsolt Kasztovszky, Ildikó Harsányi, Raghunath Acharya, Pradeep K. Pujari, Molnár Mihály, Mihaly Braun, Nahum Shabi, Olga Girshevitz, Hanoch Senderowitz

**Affiliations:** † Department of Chemistry, Bar-Ilan University, Ramat-Gan5290002, Israel; ‡ Toolmarks and Materials Lab, Israel Police HQ, Jerusalem9720045, Israel; § Laboratory for Ion Beam Interactions, Division of Experimental Physics, Rud̵er Bošković Institute, Bijenička cesta 54, ZagrebHR-10000, Croatia; ∥ Department of Physics, University of Helsinki, P.O. Box 43, HelsinkiFI-00014, Finland; ⊥ Centre for Energy Research, Konkoly-Thege Miklós út 29-33, Budapest1121, Hungary; # Radiochemistry Division, BARC, Trombay, Mumbai400085, India; ¶ International Radiocarbon AMS Competence and Training Center, ATOMKI, Debrecen4026, Hungary; ∇ Laboratory of Climatology and Environmental Physics (ICER), ATOMKI, Debrecen4026, Hungary; ○ Bar Ilan Institute of Nanotechnology and Advanced Materials, Bar-Ilan University, Ramat-Gan5290002, Israel

## Abstract

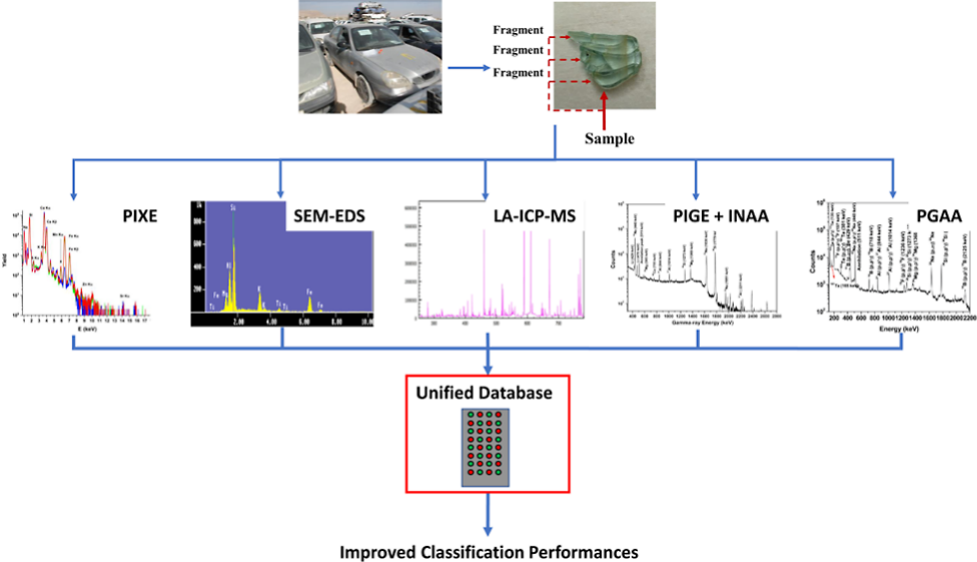

Glass fragments found in crime scenes may constitute important forensic evidence when properly analyzed, for example, to determine their origin. This analysis could be greatly helped by having a large and diverse database of glass fragments and by using it for constructing reliable machine learning (ML)-based glass classification models. Ideally, the samples that make up this database should be analyzed by a single accurate and standardized analytical technique. However, due to differences in equipment across laboratories, this is not feasible. With this in mind, in this work, we investigated if and how measurement performed at different laboratories on the same set of glass fragments could be combined in the context of ML. First, we demonstrated that elemental analysis methods such as particle-induced X-ray emission (PIXE), laser ablation inductively coupled plasma mass spectrometry (LA-ICP-MS), scanning electron microscopy with energy-dispersive X-ray spectrometry (SEM-EDS), particle-induced Gamma-ray emission (PIGE), instrumental neutron activation analysis (INAA), and prompt Gamma-ray neutron activation analysis (PGAA) could each produce lab-specific ML-based classification models. Next, we determined rules for the successful combinations of data from different laboratories and techniques and demonstrated that when followed, they give rise to improved models, and conversely, poor combinations will lead to poor-performing models. Thus, the combination of PIXE and LA-ICP-MS improves the performances by ∼10–15%, while combining PGAA with other techniques provides poorer performances in comparison with the lab-specific models. Finally, we demonstrated that the poor performances of the SEM-EDS technique, still in use by law enforcement agencies, could be greatly improved by replacing SEM-EDS measurements for Fe and Ca by PIXE measurements for these elements. These findings suggest a process whereby forensic laboratories using different elemental analysis techniques could upload their data into a unified database and get reliable classification based on lab-agnostic models. This in turn brings us closer to a more exhaustive extraction of information from glass fragment evidence and furthermore may form the basis for international-wide collaboration between law enforcement agencies.

## Introduction

There is a heavy burden on forensic experts to process forensic evidence and extract sufficient pieces of information to help investigations in solving crimes. Of the many types of forensic evidence, the analysis of glass fragments is widely utilized worldwide in cases such as homicides, hit-and-run incidents, kidnappings, robberies, and breaks and entries. Glass fragments can link broken objects to a crime scene, a victim and a suspect, and help answer questions about how, when, and what happened.^1−4^ Glass fragments easily attach to clothes, shoes, hair, skin, or other objects,^5−7^ thus presenting a large forensic potential.

To date, there are several methods for analyzing glass specimens, including refraction index (RI),^8−11^ physical attribute matching,^12^ and electron microscopy.^13−15^ However, these methods suffer from several deficiencies as previously discussed.^16^ Another analytical approach to study glass fragments, which is gaining popularity among law enforcement agencies,^17,18^ is based on elemental analysis. Elemental analysis techniques include, but are not limited to, laser ablation inductively coupled plasma mass spectrometry^19−24^ (LA-ICP-MS), particle-induced X-ray emission^16,25^ (PIXE), scanning electron microscopy with energy-dispersive X-ray spectrometry (SEM-EDS), particle-induced Gamma-ray emission^26,27^ (PIGE), instrumental neutron activation analysis (INAA), and prompt-gamma activation analysis (PGAA). In-depth description of the different methods with emphasis on forensic analysis could be found in several excellent reviews.^28,29^

Presently, there are several well-known standard test methods for the forensic analysis of glass fragments, including ASTM E1967 for RI of glass, E2926 for comparison of glass specimens using micro X-ray fluorescence (μ-XRF), and E2927 for the determination of trace elements in soda lime glass samples using LA-ICP-MS. These standards are constantly under scrutiny and improvement. For example, Corzo et al.^30^ found that E2926 comparison criteria developed for μ-XRF systems are no longer appropriate for newer, more sensitive μ-XRF systems equipped with silicon drift detectors (SDDs) and high-intensity X-ray optics. As a remedy, they suggest to increase the number of fragments collected and to modify the recommended comparison criteria. In doing so, they managed to reduce false exclusions from 23 to 2%. Similarly, Lambert et al.^31^ evaluated the new CFGS2 calibration standard, the comparison criterion recommended by the ASTM E2927 method, and provided a quantitative determination of the strength of evidence found using likelihood ratio (LR). It was observed that high LR value is obtained from glass that originated from the same windowpane, while glass that originated from different vehicles produced low LR.

To examine the feasibility of constructing a database of glass fragment measurements, Corzo et al.^32^ performed a blind test experiment across 17 laboratories using RI measurements and elemental analysis with μ-XRF and laser-induced breakdown spectroscopy (LIBS). An overall >92% correct association rate was reported for each of the three techniques, and several conclusions regarding the feasibility of the database were drawn, which include the following: (1) RI databases are readily available but not widely utilized within the forensic community. (2) It is not currently feasible to create an XRF database due to inherent signal variations among instrumental configurations. (3) There is no standard methodology for LIBS, thus preventing the establishment of a database.

The data emerging from the analysis of glass fragments could be used for two purposes, namely, association and classification. Association determines whether two specimens originate from the same source. Thus, successful association requires an exhaustive search for a matching specimen, measured by the same experimental setup. However, relevant specimens with known origin are not always available. Classification on the other hand classifies a specimen into one of the pre-determined sources (i.e., classes) and is best performed using one of many machine learning (ML) algorithms. Classification matches one-to-many (unlike association that matches one-to-one); thus, it requires a dataset of specimens per class. While classification models are less specific (one-to-many), they can be applied in multiple cases without gathering and measuring samples for a new dataset. Typically, association is more relevant in the forensic context than classification, yet both approaches may find usage, depending on the forensic question at hand.

The results of elemental analysis have been used to derive ML-based models for the classification of glass fragments.^1−3,33−39^ Thus, Tallon–Ballesteros and Riquelme^40^ used several ML algorithms such as random forest (RF), Bayes classifiers, artificial neural networks (ANNs), and nearest-neighbor methods to classify glass fragments into six classes based on their elemental composition. Similarly, Park and Carriquiry^41^ determined that better associations between the specimen and source could be obtained with RF and Bayesian Additive Regression Trees than with other algorithms. In that work, association was based on the composition of 18 elements measured by LA-ICP-MS. In a subsequent paper, Park and Tyner^18^ deduced that ML methods correctly handle non-uniformly distributed data as well as correlated data.

Recently, Kaspi et al.^16^ have used the results of PIXE-based elemental analysis performed on 96 glass fragments taken from the windshields of 13 car models from 10 car manufacturers to develop an RF-based classification model achieving an overall success rate of ∼85%. This work was subsequently extended to deriving models based on PIXE measurements made at three different laboratories. It was found that differences in measured values due to variability in equipment or lack of standardization do not necessarily lead to poorer models, provided that each set of data is normalized with respect to itself. In fact, models derived on the combined data performed as well as the best lab-specific model.^25^ However, this work only considered PIXE-based measurements.

Our ultimate goal is to assemble a global database of glass fragments from samples measured by various techniques at different laboratories, combine it with a set of ML-based tools and models, and make it accessible to as many laboratories as possible. Under this scenario, any laboratory could optimize its classification performances without investing additional resources (time or money) simply by uploading data pertaining to a specific specimen and classifying it using previously derived, technique-agnostic models. For this purpose, we first subjected the same set of glass fragments considered in our previous works^16,25^ to elemental analysis by multiple techniques including LA-ICP-MS, PGAA, PIGE, INAA, and SEM-EDS to develop lab-specific models and subsequently derived a set of rules for the successful combinations of data from different laboratories. We demonstrate that following these rules gives rise to improved models, and conversely, violating them leads to poor-performing models.

## Materials and Methods

Recent works^16,25^ demonstrated the usefulness of a workflow designed to derive ML-based models for the reliable classification of glass fragments’ original car manufacturer. This workflow is based on the elemental composition of fragments taken from cars’ windshields measured by the PIXE technique. To construct this workflow, glass fragments collected by the Israeli DIFS were distributed to three labs specializing in PIXE-based analysis, namely, Bar-Ilan Institute of Nanotechnology (BINA), Laboratory for Ion Beam Interactions, Rud̵er Bošković Institute (RBI), and the Department of Physics, Accelerator Laboratory, University of Helsinki (UH). Measurements performed by the individual labs produced classification models with good performances (>80%) and their unification afforded models with performances matching those of the best-performing, lab-specific model.

The favorable performances obtained by unifying PIXE-based measurements from different laboratories testify to the potential usefulness of a large, glass fragment measurements database. However, to increase the scope and diversity of the database, for example, by allowing it to incorporate data from multiple laboratories, it is important to expand the database to include various techniques for elemental composition analysis. To this end, in this work, we combine measurements of the same specimens from laboratories using different techniques for elemental analysis.

We distributed the previously measured and analyzed samples to four additional laboratories that rely on elemental analysis techniques other than PIXE, specifically Institute for Nuclear Research, Hungary, ATOMKI (LA-ICP-MS), Israeli Police Force, Israel, DIFS (SEM-EDS), Bhabha Atomic Research Centre, Radiochemistry Division, India, BARC (PIGE, INAA), and Budapest Neutron Centre, Centre for Energy Research, Hungary, BNC (PGAA). Each method has a different measurement accuracy as well as sensitivity to major, minor, and trace elements. Table 1 displays the details of the experimental setups of the four additional laboratories as well as the PIXE-based laboratories.^42−44^

**Table 1:**
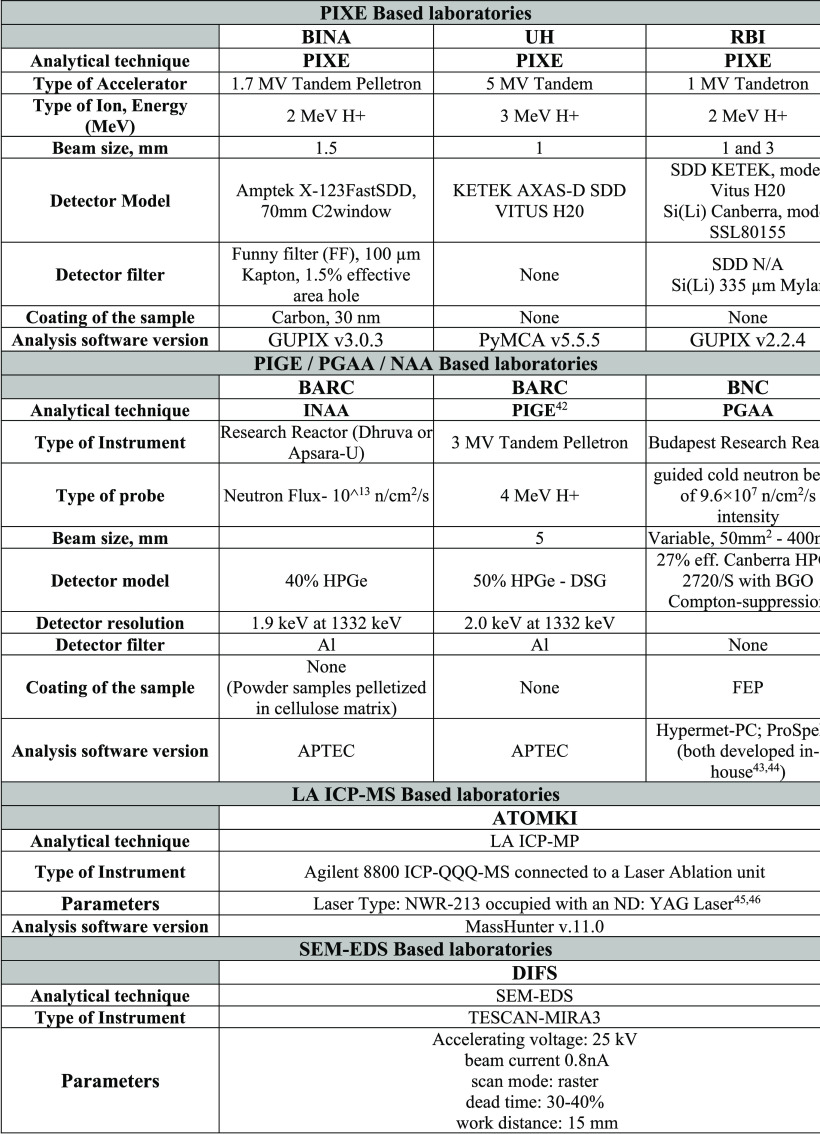
Details of the Participating Laboratories’ Experimental Setups

**Figure 1:**
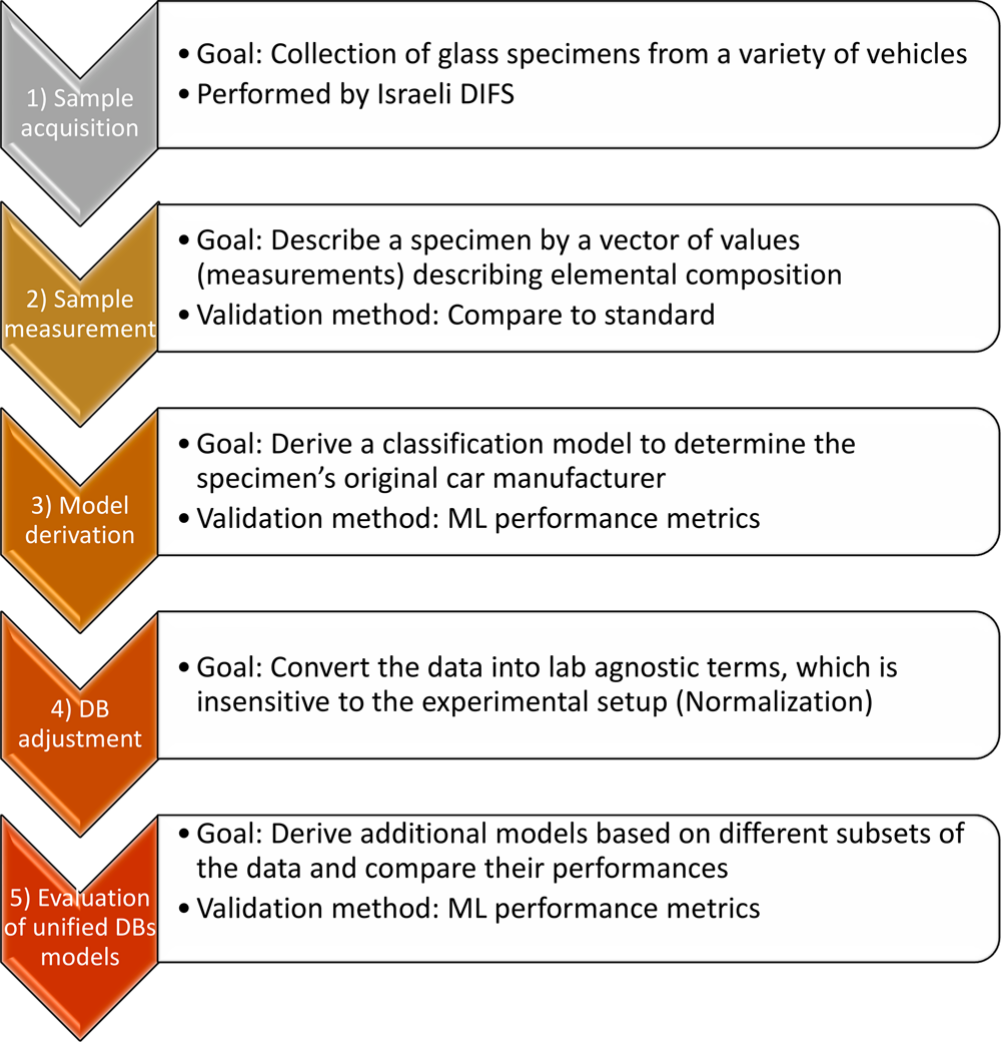
Methodology used in this work. First three stages, (1) sample acquisition, (2) sample measurement, and (3) model derivation were used in our first work^16^ and steps (4) database (DB) adjustment and (5) evaluation of unified DB models were introduced in the second work.^25^

The workflow employed in this work largely follows the one used in our previous works^16,25^ (Figure 1). In sample acquisition, 48 glass specimens from 13 different car models from 10 car manufacturers were collected and recorded. The same specimens that were previously measured by PIXE were now provided to all labs for analysis by other techniques.

Prior to specimen processing, each laboratory had to evaluate the analytical performance of the different methods by measuring the Standard Reference Materials NIST-620 and NIST-610. The experimental setups were optimized so as to reproduce some or all known concentrations in these standards, namely, Na, Mg, Al, Si, K, Ca, S, Cl, Ti, Fe, Cr, Sn, and Mn. This stage is a prerequisite for any future analysis since it validates the ability of the technology to correctly measure glass specimens.

In sample measurement, each sample was analyzed for its elemental composition, and each element concentration was expressed in units of parts per million (ppm). Elemental composition measurements require homogeneity which was previously demonstrated.^16^ One factor that may compromise sample homogeneity is corrosion, known to occur on glass surfaces with time. This directly affects the concentration of Na on the surface but not in the bulk. To quantify this effect, analytical methods that used whole samples (SEM-EDS, PIXE, and PIGE) measured them in two orientations corresponding to the surface and bulk (specimen that had a smooth surface likely originate from the surface of the glass, while those with ruptured surfaces likely originate from the bulk of the glass). The previous study reported a negligible effect of corrosion on the performances of classification models.^16^ INAA and LA-ICP-MS are bulk analysis techniques; thus, any surface effects such as corrosion would be insignificant.

Naturally, different analytical techniques measure different sets of elements, while some elements fall below the limit of detection (LOD). As an example, the Sn glass bulk content is most often null or below the LOD of several of the used analytical techniques. Sn detection usually reveals the use of float glass that presents a thin surface layer containing Sn. Thus, for dataset uniformity, Sn was not included in the following analysis. Table 2 summarizes the number of elements measured by each laboratory and technique. Following measurements, each specimen was characterized by a vector of numbers, each corresponding to the concentration of a different element. We refer to these numbers as the sample’s features or features vector. The compilation of all feature vectors of all samples is referred to as the dataset.

**Table 2:** Participating Laboratories, Their Analytical Techniques, and the Dimensionality of Their Feature Vectors

lab name	technique	no. of features	no. of samples	no. of samples per technique
**BINA**	PIXE	18	92	284
**RBI**		10	96	
**UH**		6	96	
**BARC**	PIGE + INAA	22^a^	48	68
**BNC**	PGAA	14	20	
**ATOMKI**	LA-ICP-MS	44	48	48
**DIFS**	SEM-EDS	7	96	96

a Elements were measured either by PIGE or by INAA. The list of elements measured by each technique is detailed in Table 4.

Initially, the data obtained from each individual lab were used to construct lab-specific classification models. To this end, each lab-specific dataset was randomly partitioned into training and test sets with a ratio of 67:33%, respectively. Then, RF parameters (see Table 3) were optimized by means of a random grid search, and the different combinations were evaluated using the recall, precision, and F1-score metrics following the three-fold cross-validation (CV) on the training set. Briefly, recall represents the ability to properly identify all of the elements in a particular class and is expressed as the ratio of correctly classified samples [true positive (TP)] to the total number of samples in the class [TP + false negative (FN)]. Precision complements recall by determining how well a class is being determined, that is, the ratio between the number of correctly classified samples (TP) and the number of all samples classified to the class of interest [(TP) + false positive (FP)]. F1-score is the harmonic mean of the precision and recall [precision*recall/precision + recall*2]. The models with the best performances were then evaluated on the test sets and the results compared to those obtained by “benchmark” models using default values for all parameters defined by the RF implementation in the Scikit Python library. Overall, no significant improvement was observed in any of the models derived for the individual laboratories (F1-score difference < 0.2), so default Scikit values were kept for all subsequent model constructions (100 trees, no max depth, min_samples_split = 2, bootstrap = True).

**Table 3:** RF Parameter Optimized in This Work

parameter	description	search span
**Num_estimators**	number of different trees in the forest	min value: 200
		max value: 1800
		step size: 200
**max_features**	the maximum allowed number of features to consider for the best possible split.	max_features = log 2(n_features) or max_features = sqrt(n_features)
**max_depth**	the maximum depth of the tree	min value: 10
		max value: 100
		step size: 10
**min_samples_split**	the minimum number of samples required to split an internal node	2, 5 or 10
**min_samples_leaf**	the minimum number of samples required to be at a leaf node	1,2 or 4
**Bootstrap**	whether bootstrap samples are used when building trees	true or false

**Figure 2:**
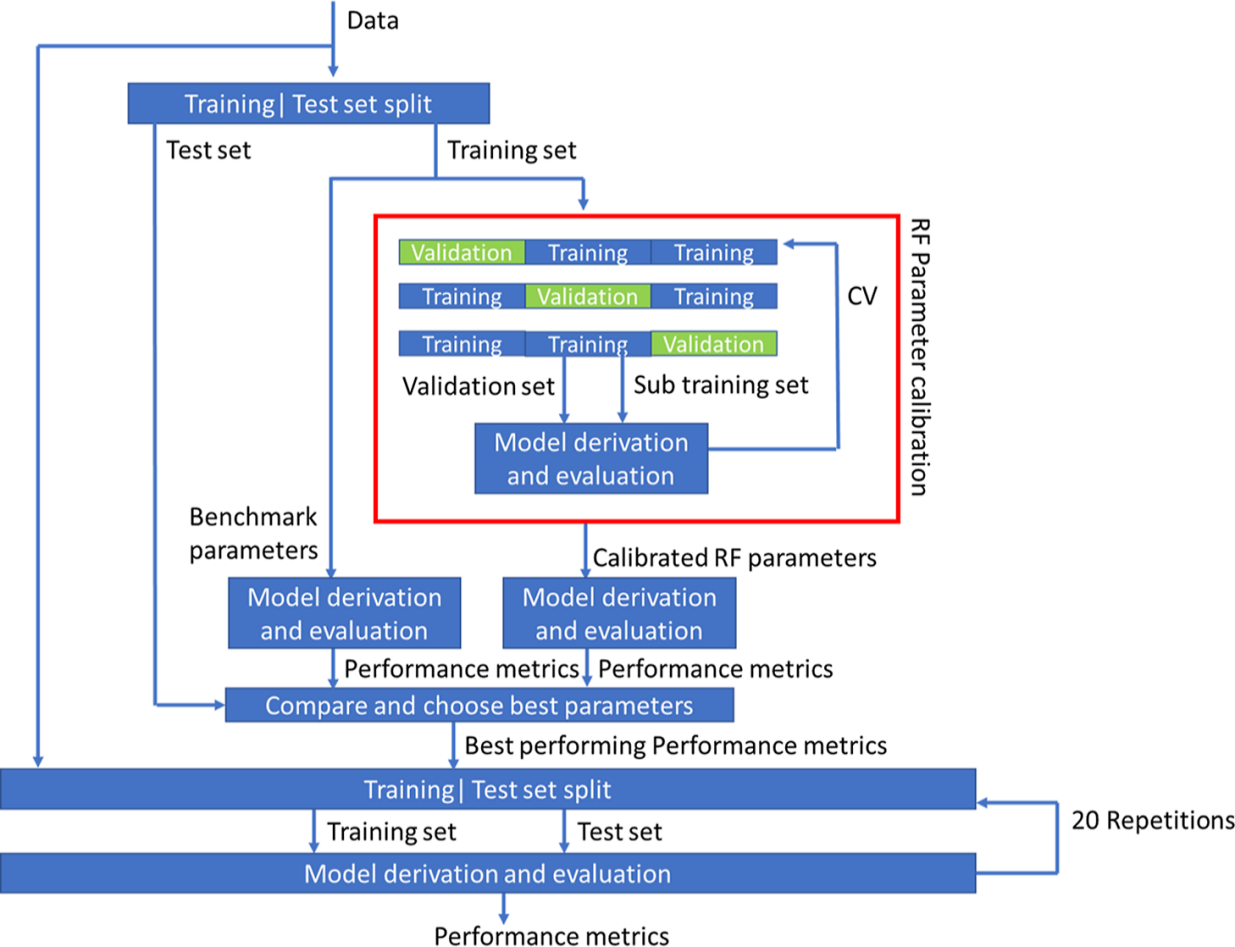
Workflow for the derivation of classification models using RF.

Having decided on optimal RF parameters, each lab-specific dataset was randomly divided into training and test sets as described above, and models were constructed from the training set and evaluated on the test sets using the precision, recall, and F1-score metrics. This process was repeated 20 times for consistency, and the results are reported as average ± SD values. A purely random model would produce performance metrics of ∼0.1 for precision, recall, and F1-score as there is approximately 0.1 probability to correctly classify a glass fragment to a car manufacturer. Therefore, a good model would be one that significantly outperforms this threshold. To ensure that the models were not over-fitted, Y-scrambling was applied to all splits, and the resulting performances were found to be similar to the theoretical values for a purely random model (data not shown). Finally, linear classification models were also derived using a support vector machine (SVM) following largely a similar approach. The entire process of model derivation and validation is depicted in Figure 2.

Having developed lab-specific models, we set up to unite the measurements from the different laboratories into a single database. This stage required a data adjustment phase where the data is converted to lab-independent units. The adjustment was performed via *Z*-score normalization according to eq 1, where *x* is the value of the measurement, μ is the mean value of the feature, and σ is its standard deviation.
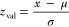
1

The rationalization behind this normalization is that different laboratories with different equipment and methodologies produce different nominal measured values for each element in the same specimen. Yet, the overall “ordering” of the specimen within the dataset should be identical across all laboratories. *Z*-score normalization addresses this by expressing specimens’ values in relation to the feature’s means and variance, thus avoiding setup-specific measurement offsets. After the normalization, the normalized datasets from the individual labs were combined to create a unified database (DB).

The combined DB (also called a unified DB) and subsets derived from it allow for the derivation of glass classification ML models based on data from multiple experimental measurements. This is the essence of evaluation of unified DB model stage (and models derived from the subsets). Importantly, investigating the causes for the different performances between models derived from different types of data will produce important insights into the elemental analysis techniques used for obtaining these data.

Finally, it is to be expected that models derived from SEM-EDS measurements will perform significantly poorer than other methods^13,15,45−47^ (a statement that is supported by the results presented in the Results section). While gradually being replaced by better methods, SEM-EDS is still in usage.^14,48^ Therefore, identifying means to improve SEM-EDS performances will have both scientific and immediate practical implications. To this end, SEM-EDS measurements for each element were replaced, one at a time, by the corresponding values obtained by PIXE measurements (used as a benchmark), and the resulting “chimeric” datasets were subjected to the above-described model derivation procedure. This approach allowed for unveiling which of the SEM-EDS-measured elements should be reanalyzed in a more accurate manner.

## Results

**Figure 3:**
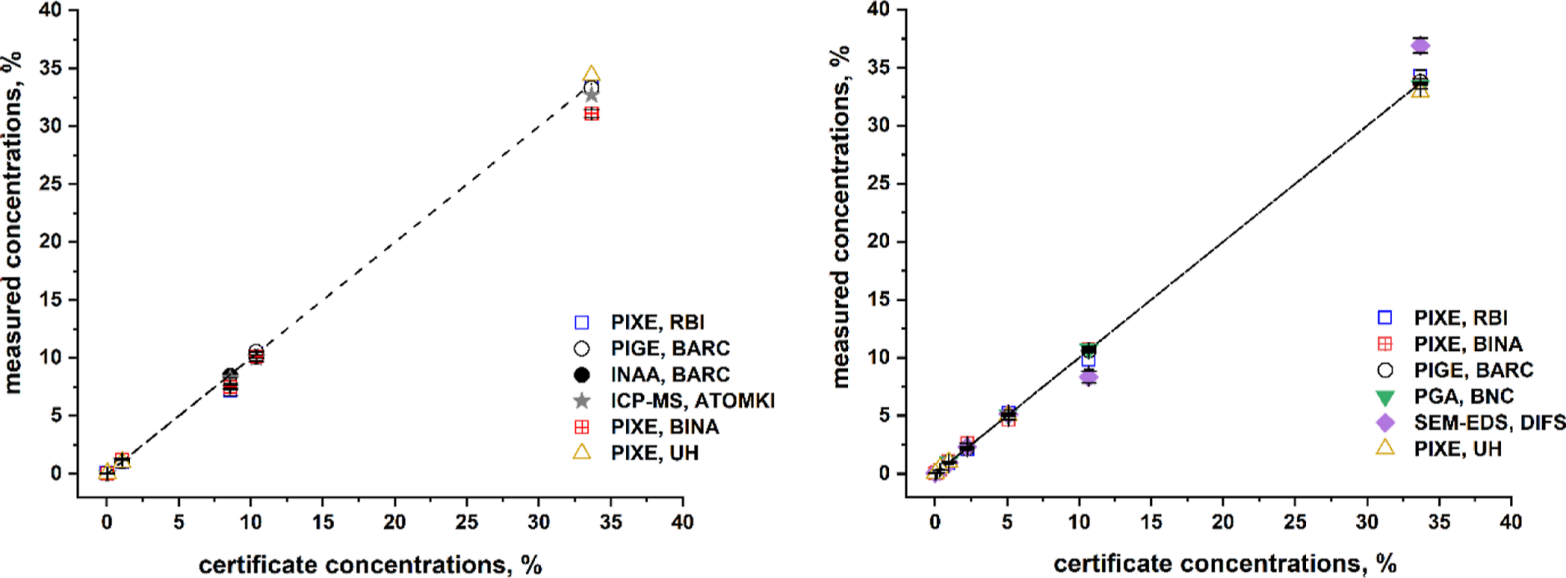
Agreement of measurements’ results between different analytical techniques from different laboratories and the known standard compositions of NIST-610 (left) and NIST-620 (right).

All participating laboratories calibrated their experimental setup to reproduce the elemental composition of the NIST-610 and NIST-620 standards (see Table 4). All techniques, except SEM-EDS, showed a good agreement with the known certified concentrations as presented in Figure 3. SEM-EDS results are significantly different from the certificate concentrations, and the sources of errors are well understood.^13,15,45−47^ These include inappropriate setup parameters such as work distance, accelerating voltage, pulse throughput, and count rate that can be the cause of poor background fit, leading to less-accurate quantification results and difficulties in deconvolution due to pile-up peaks. In addition, samples’ inhomogeneity, rough surface, and charging effect are reasons for non-normalized concentrations >110% and poor fit of energy background.

**Table 4:** Concentrations, Uncertainty, and LOD for NIST-610 and NIST-620 as Obtained by the Different Laboratories

	SRM#620, certificate	PIXE, RBI			PIXE, BINA			PIGE, BARC			PGAA, BNC			SEM-EDS, DIFS		
element	conc, %	conc, %	U(+/–1s)	LOD, ppm	conc, %	U(+/–1s)	LOD, ppm	conc, %	U(+/–1s)	LOD, ppm	conc, %	U(+/–1s)	LOD, wt %	conc, %	U(+/–1s)	LOD, wt %
Na	10.67	9.8	0.8	130	10.71	0.04	214	10.6	0.1	112	10.7	0.2	0.070	8.3	0.5	0.100
Mg	2.22	2.09	0.06	103	2.64	0.01	90	2.18	0.09	560	N/A		0.480	2.30	0.03	0.100
Al	0.95	0.91	0.06	80	1.05	0.01	182	0.96	0.03	265	1.00	0.05	0.794	0.900	0.006	0.100
Si	33.69	34.3	0.5	66	33.71	0.02	157	33.8	0.3	467	33.4	0.2	0.400	36.91	0.6	0.100
S	0.112	0.10	0.01	83	0.079	0.005	137	N/A			N/A			N/A		0.100
K	0.340	0.32	0.02	79	0.290	0.001	10	N/A			N/A			0.25	0.01	0.100
Ca	5.08	5.2	0.2	66	4.63	0.02	24				5.1	0.1	0.350	5.12	0.08	0.100
Ti	0.011	0.010	0.001	10	0.007	0.001	6							N/A		0.100
Fe	0.030	0.033	0.001	4	0.021	0.001	8							N/A		0.100

	SRM#610, certificate	PIXE, RBI			PIXE, BINA			PIGE, BARC			INAA, BARC			LA-ICP-MS, BNS		
element	conc, %	conc, %	U(+/–1s)	LOD, ppm	conc, %	U(+/–1s)	LOD, ppm	conc, %	U(+/–1s)	LOD, ppm	conc, %	U(+/–1s)	LOD, ppm	conc, %	U(+/–1s)	LOD, ppb
Na	10.39	10.4	0.4	348.2	10.1	0.1	277	10.5	0.4	112	10.1	0.1	56	10.0	0.3	
Al	1.059	1.04	0.02	101.8	1.2	0.1	173	1.07	0.06	265	N/A		1	1.1	0.2	4.000
Si	33.66	33.5	0.3	266.3	31.1	2.7	234	33.3	0.4	467	N/A			32.7	0.900	10.000
Ca	8.58	7.2	0.1	81.3	7.5	0.8	49				8.5	0.3	33	8.2	0.4	
Ti	0.044	0.039	0.003	25.1	0.033	0.003	15				N/A			0.043	0.007	0.396
Cr	0.042	0.037	0.003	20.1	0.030	0.006	18				N/A		1	N/A		
Mn	0.046	0.040	0.003	23.1	N/A						N/A			0.049	0.001	0.200
Fe	0.046	0.044	0.001	24.1	0.031	0.009	22				0.045	0.001	157	0.045	0.000	3.000
Co	0.039	0.036	0.060	27.7	N/A											
Ni	0.046	0.044	0.001	28.1	0.028	0.004	26				N/A			N/A		
Cu	0.044	0.044	0.001	29.2	N/A						N/A			0.043	0.000	0.122
Zn	0.043	0.048	0.002	22.0	N/A						0.046	0.003	2	0.046	0.001	0.134
As	0.034	0.030	0.002	33.0	0.028	0.004	33				N/A			0.032	0.000	
Se	0.012	0.011	0.002	24.6	0.005	0.001	31				N/A			N/A		
Rb	0.043	0.042	0.003	30.2	0.025	0.006	79				0.042	0.002	2	0.043	0.001	0.100
Sr	0.052	0.046	0.006	29.3	0.026	0.005	120				N/A			0.052	0.002	0.060

Following the successful calibration, each lab analyzed the dataset of glass fragments, and the resulting elemental compositions were used as features for the derivation of lab-specific ML-based classification models using the RF and SVM algorithms. The results are presented in Table 5 and show that in almost all cases, RF-based models outperformed the SVM-based models.

**Table 5:** Performances of Lab-Specific Models on Test Sets^a^

	RF			SVM		
lab group	precision	recall	F1-score	precision	recall	F1-score
**BINA**	0.84 ± 0.05	0.88 ± 0.05	0.83 ± 0.05	0.81 ± 0.10	0.77 ± 0.07	0.76 ± 0.08
**RBI**	0.84 ± 0.05	0.87 ± 0.05	0.83 ± 0.05	0.81 ± 0.08	0.79 ± 0.06	0.77 ± 0.07
**UH**	0.65 ± 0.08	0.66 ± 0.13	0.61 ± 0.10	0.56 ± 0.10	0.60 ± 0.07	0.55 ± 0.08
**BARC**	0.92 ± 0.06	0.87 ± 0.10	0.89 ± 0.08	0.36 ± 0.13	0.39 ± 0.07	0.35 ± 0.09
**BNC**	0.52 ± 0.25	0.48 ± 0.26	0.49 ± 0.25	0.48 ± 0.18	0.56 ± 0.17	0.51 ± 0.19
**ATOMKI**	0.79 ± 0.09	0.76 ± 0.12	0.76 ± 0.10	0.66 ± 0.14	0.66 ± 0.10	0.63 ± 0.12
**DIFS**	0.51 ± 0.09	0.52 ± 0.11	0.48 ± 0.10	0.56 ± 0.08	0.58 ± 0.06	0.53 ± 0.07

a Results are expressed as means ± SD over 20 random splits.

The resulting RF models were also analyzed for the most significant features. Table 6 presents the elements that appeared among the 10 topmost important features in at least 18 out of 20 models, and Table 7 presents the agreement between different labs on feature importance. It is evident that important features chosen by most laboratories (e.g., Al, Fe, and Ca) are either not chosen by models derived from BARC measurements or not measured at all (e.g., K, Ti, and Mn). Of note, elements that were previously reported to be discriminating elements, such as Sr and Zr, were not chosen to important for classification in this dataset.^32,49^

**Table 6:** Top 10 Features, Most Frequently Selected by the RF Algorithm for Each Individual Lab^a^

order of importance	BINA/PIXE (18)	RBI/PIXE (10)	UH/PIXE (6)	BARC/PIGE + INAA (22)	BNC/PGAA (14)	ATOMKI/LA-ICP-MS (44)	DIFS/SEM-EDS (7)
**1**	K	Ca	Fe	Ce	K	Ca	Ca
**2**	Al	Fe	K	Sc	Al	La	Al
**3**	Ti	Mn	Si	Zn	Gd	Fe	K
**4**	Fe	K	Al	Eu	Ti		Fe
**5**	Mn	Mg	Ca	Zr	Cl		Si
**6**	Ca	Al	Ti	La	B		Mg
**7**	Zn	Si		Co	Si		Na
**8**		Cr		Rb	Fe		
**9**		S					
**10**		Na					

a The maximal number of measurable features for each lab is given in parenthesis.

**Table 7:** Agreement between Labs on Features’ Importance^a^

elements	BINA/PIXE	RBI/PIXE	UH/PIXE	BARC/PIGE + INAA	BNC/PGAA	ATOMKI/LA-ICP-MS	DIFS/SEM-EDS
**K**	X	X	X	*	X		X
**Al**	X	X	X		X		X
**Ti**	X	*	X	*	X		*
**Fe**	X	X	X		X	X	X
**Mn**	X	X	*	*			*
**Ca**	X	X	X		*	X	*
**Mg**		X	*				X
**Zn**	X	*	*	X	*		*
**Si**		X	X		X		X
**Cr**		X	*		*	*	*
**S**		X	*	*	*	*	*
**Na**		X	*		X		X
**Ce, Zr, Rb, Eu**	*	*	*	X	*		*
**Sc, Co**	*	*	*	X	*	*	*
**La**	*	*	*	X	*	X	*
**Gd, B**	*	*	*	*	X		*
**Cl**	*	*	*	*	X	*	*
**Pr, As, Bi**	*	*	*	*	*		*

a Cells with X represent features that were measured and found among the 10 topmost important features. Empty cells represent features that were measured but not determined to be within the 10 topmost important features. Cells with * represent elements that were not measured by the specific lab.

All labs demonstrated their ability to produce models with performances better than those expected from random classification, and therefore, their measurements were combined into a unified dataset of 496 measurements. To this end, data from the individual labs was first *z*-score-normalized and subsequently combined with data from the other laboratories. Then, for the purpose of consistency, features (e.g., elements) that were not measured by all laboratories were discarded, leaving only four features (Si, Al, Ca, and Fe). This step, while necessary, may well compromise the performances of subsequent models as was already noted by Kaspi et al.^25^ This is particularly true when combining different techniques that potentially measure a significantly different number of elements. Indeed, a significant loss of potential information was observed for BARC that uses PIGE and INAA measurements and retained only 18% of its original set of features.

The unified dataset was randomly divided 20 times into training and test sets in a 67:33 ratio, and RF-based classification models were derived from the training set and evaluated on the test set. The resulting test set-based metrics are 0.67 ± 0.03, 0.68 ± 0.03, and 0.66 ± 0.04 for recall, precision, and F1-score, respectively. We hypothesized that discarding data from laboratories whose individual model performances were significantly lower than the rest would improve the performances of the model derived from the combined dataset. To test this hypothesis data from laboratories, performance values < 0.7 were discarded (BNC, DIFS, and UH). Models derived from the remaining 284 measurements represented by six features (Si, Mg, Na, Al, Ca, and Fe) indeed demonstrated improved performances with recall, precision, and F1-score values of 0.79 ± 0.03, 0.82 ± 0.03, 0.79 ± 0.03, respectively.

**Figure 4:**
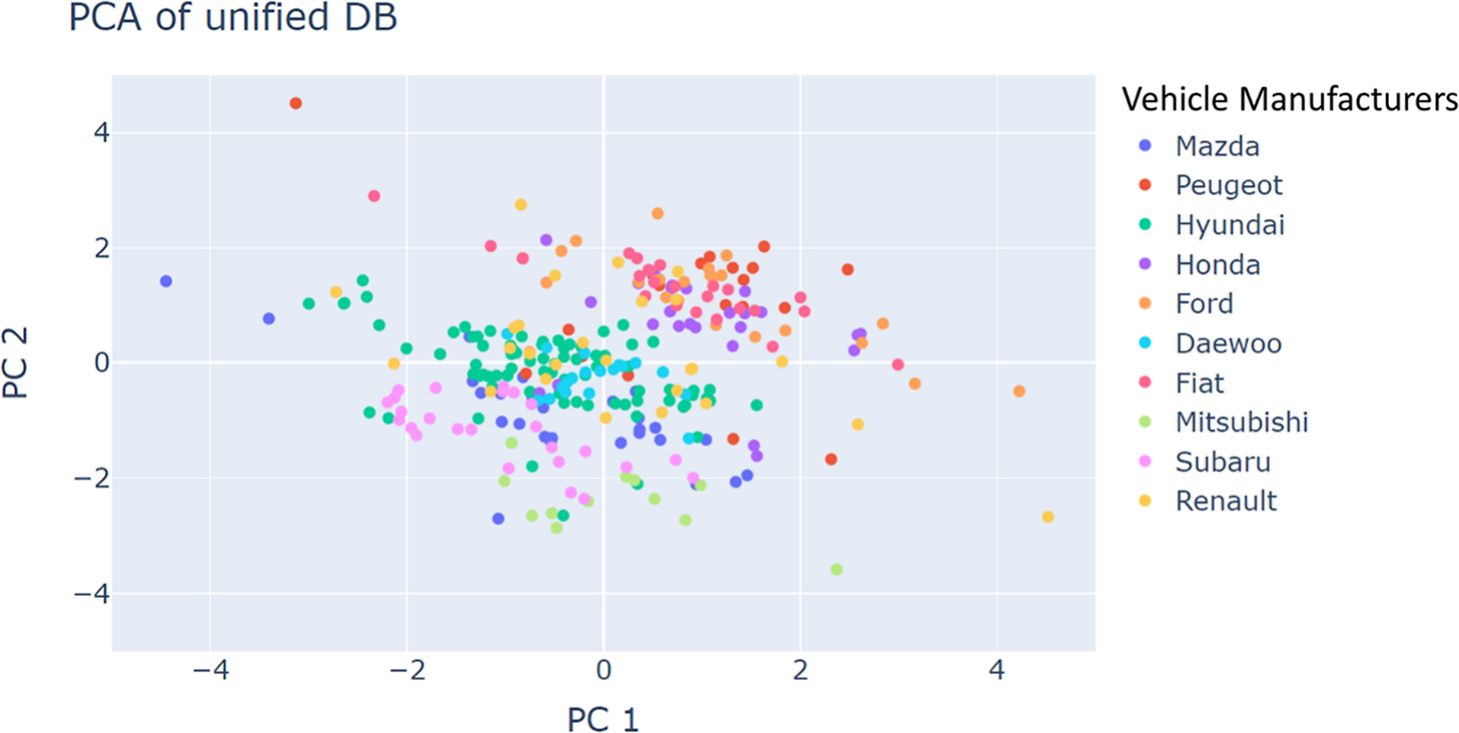
PCA of the unified and filtered dataset (284 glass fragments each characterized by six elements) color coded according to vehicle manufacturers.

**Figure 5:**
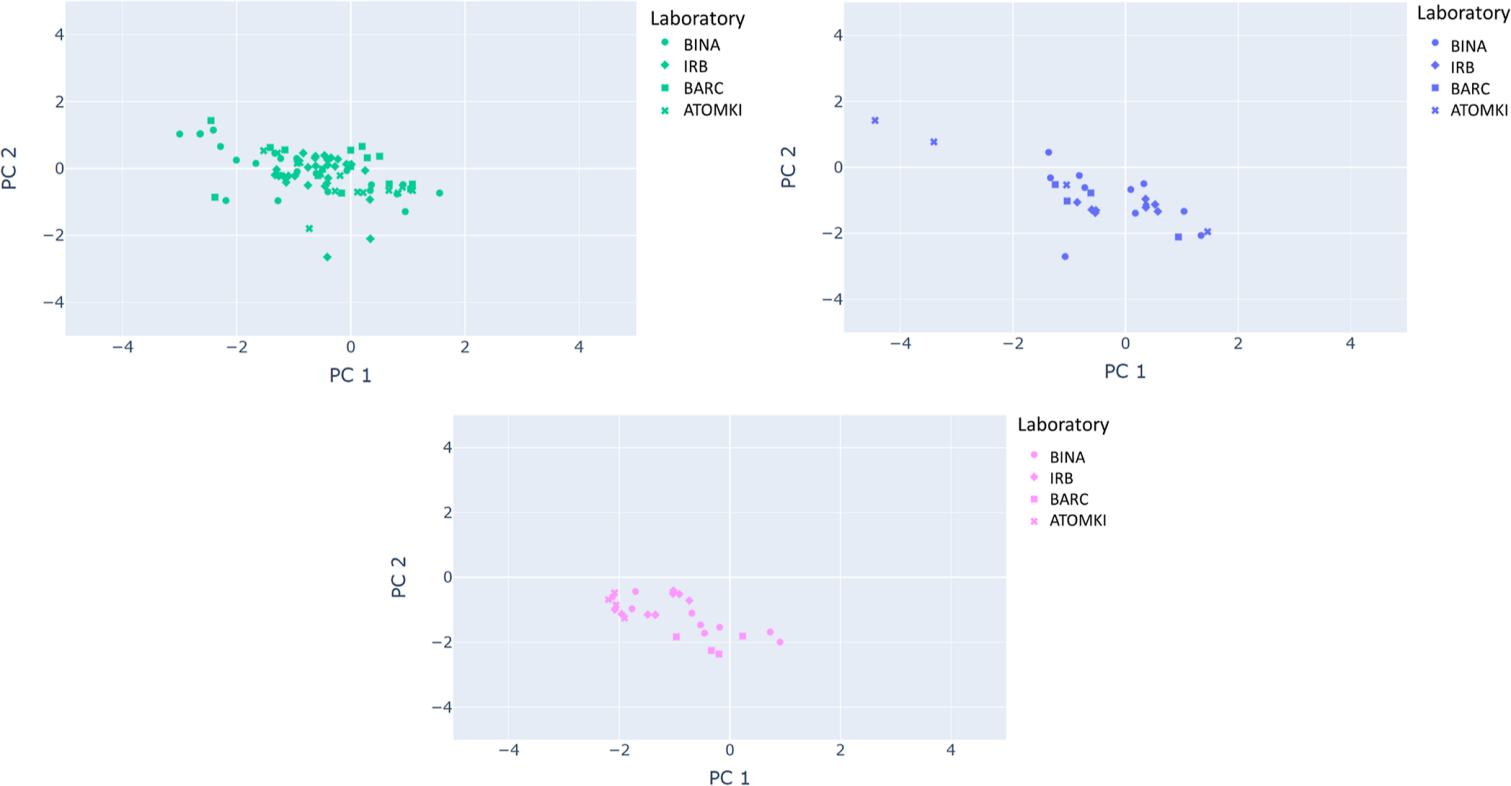
PCA of glass fragments taken from the same car manufacturer and analyzed by the four laboratories that survived the filtration process. The top, middle, and bottom panels represent Hyundai, Mazda, and Subaru, respectively.

Using principal component analysis (PCA) as a dimensionality reduction method, we have previously demonstrated that the overall shape of the distribution of car manufacturers in the space of the measured elements is maintained across all laboratories that used the PIXE technique.^25^ To test whether this observation still holds across elemental compositions measured by other techniques, we have subjected the unified and filtered dataset (284 glass fragments each characterized by six elements) to a similar analysis. The results are presented in Figure 4 and demonstrate that following labwise normalization, samples belonging to the same vehicle manufacturer are located in the same region of the PC plot, irrespective of the lab. Figure 5 shows that the distribution of various lab measurements has a similar shape and location per car manufacturer.^25^

Yet, even after discarding data from several laboratories, the performances of the resulting models only matched those of the worst lab-specific models. Thus, several combinations of data from different labs were attempted in order to identify the best (and worst) ones. Each combination was created by unifying the normalized measurements of the individual techniques while keeping the features common to them. The results of these experiments are presented in Table 8. It is observed that several combinations (e.g., PIXE/LA-ICP-MS, and LA-ICP-MS/SEM-EDS) improve model performances beyond the performances of the lab-specific models, while others (e.g., PIGE + INAA/SEM-EDS) degrade model performances to below the performances of the specific models. All performance differences caused by the various combinations were determined to be statistically significant using Student’s *t*-test with α = 0.05.

**Table 8:** Performances of Models Derived from Combinations of Measurements Performed by Different Techniques on Test Sets^a^

	PIXE	PIGE + INAA	LA-ICP-MS	SEM-EDS
**PIXE**	P: 0.87 ± 0.04	P: 0.78 ± 0.05	P: 0.92 ± 0.02	P: 0.81 ± 0.04
	R: 0.90 ± 0.03	R: 0.81 ± 0.04	R: 0.93 ± 0.02	R: 0.84 ± 0.03
	F1: 0.87 ± 0.05	F1: 0.77 ± 0.05	F1: 0.92 ± 0.03	F1: 0.81 ± 0.05
**PIGE + INAA**		P: 0.92 ± 0.06	P: 0.86 ± 0.06	P: 0.49 ± 0.06
		R: 0.87 ± 0.10	R: 0.83 ± 0.09	R: 0.50 ± 0.09
		F1: 0.89 ± 0.08	F1: 0.83 ± 0.07	F1: 0.46 ± 0.07
**LA-ICP-MS**			P: 0.79 ± 0.09	P: 0.88 ± 0.06
			R: 0.76 ± 0.12	R: 0.89 ± 0.06
			F1: 0.76 ± 0.10	F1: 0.86 ± 0.07
**SEM-EDS**				P: 0.51 ± 0.09
				R: 0.52 ± 0.11
				F1: 0.48 ± 0.10

a Rows and columns represent the different contributors to the combination, and the diagonal cells represent the performance of lab-specific models.

While unsurprisingly SEM-EDS measurements^13,15,45−47^ produce classification models that perform consistently worse than all other models, it was gratifying to see that combining SEM-EDS measurements with either PIXE or LA-ICP-MS measurements improves the performances of the resulting model.

Next, we tested whether SEM-EDS measurements could give rise to models with good performances if complemented by more accurate measurements for specific elements (features for ML). In this case, we focused on PIXE as the complementing method for two reasons: (1) PIXE and SEM-EDS share many common elements and (2) PIXE measurements afforded models with good performances. Indeed, we found that by gradually replacing the SEM-EDS data with the PIXE data, model performances improve, and the most significant improvement was achieved by replacing the measurements of Fe and Ca, where an improvement of ∼15% was observed in all the three metrics. Two main reasons could be considered for this significant improvement: (1) the range of concentrations of Fe in the glass specimens is in many cases close to the LOD of SEM-EDS, which is roughly 2–3 orders of magnitude higher than for PIXE and (2) an overlap of Ca peaks with Sn peaks causes inaccurate determination of Ca concentrations by SEM-EDS. Regarding the discrepancies of Ca results by SEM-EDS that were attributed to the spectral interference of Sn, indeed this might be the case if Sn was not identified as an element present in the sample. The spectral resolution could be one of the problems, as well as the high background and/or a high count rate, thus additionally increasing the detection limit, especially if the counting dead time was high (reported in this work as 30–40%, Table 1). On the other hand, the detection limits are orders of magnitude lower for PIXE than for SEM-EDS; hence, the correct peak assignment of trace elements is achievable.

## Discussion

In a previous report, we have demonstrated the benefits of combining data from different laboratories, albeit measured by the same analytical technique (PIXE), to derive glass classification models that either match or even suppress the performances of models derived from lab-specific data.^25^ However, the performances of models derived from the unified database in the present work are not in par with this previous observation. To further investigate this point, we address two follow-up questions: (1) Why were the unified model performances less than expected? (2) How can model performances be improved?

A very likely reason for the decrease in performances for models derived from the unified database is the removal of features not common to all laboratories. To examine the influence of this reduction while disregarding potential effects resulting from the unification of the datasets, classification models were derived from individual laboratories’ datasets using only features that were included in the unified DB. Table 9 presents a comparison between the performances of models derived from a dataset with all of the original features and those from the common features subset. It is evident that BARC is the lab that is affected the most from the feature reduction (model performances decreased from 90 to 30–40%), while other lab experience a lesser impact. Indeed, as noted above, upon measurements, unification BARC retained only 18% of the original number of measured elements.

**Table 9:** Comparison of the Performances of Models Derived from All Features Measurable by the Corresponding Analytical Technique and from the Subset of Common Features Characterizing the Unified Database^a^

	full feature list	subset feature list (6 features)
**PIXE/BINA and RBI(10 features)**	P: 0.87 ± 0.04	P: 0.81 ± 0.03
	R: 0.90 ± 0.03	R: 0.83 ± 0.04
	F1: 0.87 ± 0.05	F1: 0.79 ± 0.04
**PIGE + INAA/BARC(22 features)**	P: 0.92 ± 0.06	P: 0.40 ± 0.13
	R: 0.87 ± 0.10	R: 0.29 ± 0.15
	F1: 0.89 ± 0.08	F1: 0.31 ± 0.14
**PGAA/BNC(14 features)**	P: 0.52 ± 0.25	P: 0.46 ± 0.17
	R: 0.48 ± 0.26	R: 0.38 ± 0.19
	F1: 0.49 ± 0.25	F1: 0.40 ± 0.18
**LA-ICP-MS/ATOMKI(44 features)**	P: 0.79 ± 0.09	P: 0.68 ± 0.11
	R: 0.76 ± 0.12	R: 0.71 ± 0.15
	F1: 0.76 ± 0.10	F1: 0.66 ± 0.12
**SEM-EDS/DIFS(7 features)**	P: 0.51 ± 0.09	P: 0.46 ± 0.06
	R: 0.52 ± 0.11	R: 0.43 ± 0.10
	F1: 0.48 ± 0.10	F1: 0.42 ± 0.07

a Performances are shown for test sets.

Focusing on BARC-derived data, we note that (1) BARC by itself produces high-performing models. (2) Models derived by BARC using the common features’ subset perform significantly worse. (3) Features selected by BARC to be important for model derivation are absent from the common features’ subset (Table 7). Taken together, we suggest that BARC produces high-quality classification models using a completely different features’ set than is used by all other methods. This in turn prevents the successful combination of BARC measurements with measurements performed by other analytical techniques. This led us to hypothesize that for a laboratory to be able to benefit from the unified DB, it is not sufficient for it to be able to properly measure samples and construct a reasonably predictive model but rather to be able to construct a reasonably predictive model using the important features as determined by other labs’ models.

Table 10 presents the overlap between the elements deemed to be important across all labs and gives rise to several insights. First, while BINA and RBI are both PIXE based, not all of the important features are shared between them; specifically, Mg, Si, Cr, S, and Na were found to be important only by one lab. This could be at least partly attributed to differences in detectors. Still, the combination of measurements from both laboratories afforded good models (Table 8).

**Table 10:** Overlap between Elements Found to Be Important across All Labs

name of laboratories	analytical techniques	common elements
ATOMKI + BARC	PIGE + INAA + LA-ICP-MS	La
BNC, BINA	PGAA + PIXE	Ti
BARC, BINA	PIGE + INAA + PIXE	Zn
BINA, RBI	PIXE	Mn
DIFS, RBI	SEM-EDS + PIXE	Mg, Na
BINA, UH	PIXE	Ti
DIFS, BINA, RBI, UH	SEM-EDS + PIXE	K, Al, Fe
DIFS, RBI, BNC, UH	SEM-EDS + PIXE + PGAA	Si
DIFS, RBI, BNC, BINA, UH	SEM-EDS + PIXE + PGAA	K, Al
ATOMKI, BINA, RBI, UH	LA-ICP-MS + PIXE	Ca
ATOMKI, BINA, RBI, BNC, DIFS, HU	LA-ICP-MS + PIXE + SEM-EDS	Fe

Furthermore, ATOMKI LA-ICP-MS shares important features with RBI (PIXE), BINA (PIXE), DIFS (SEM-EDS), and BNC (PGAA) on the one hand and with BARC (PIGE and INAA) on the other hand, thus being the only laboratory with measurements that could be favorably combined with measurements from all other labs (Tables 7, 8, and 10).

Thus, in general, combining measurements from any two labs that share important features leads to models which are significantly better than the lower performing model of the two labs and are only slightly worse than the higher performing model (Tables 7 and 8).

Of note, the best lab-specific measurements to be combined turned out to be those based on PIXE and LA-ICP-MS. A model derived on the combined data outperformed the PIXE-based model by ∼2–5% and the LA-ICP-MS model by ∼15% (Table 8). Those two analytical techniques share two important features, Fe and Ca, yet at the same time complement each other.

In summary, this work provides preliminary evidence that measurements performed on glass fragments from cars’ windshields by different analytical techniques could, under certain circumstances, be favorably combined into reliable classification models. Yet, the overall relevance of such models in the context of forensic analysis, and, particularly, in comparison with establishing associations or a common source with a reference material, should be discussed. Association can unequivocally identify the exact source of the fragment, however, only if a large enough set of known samples is available. Moreover, reliable association requires the sample of interest and the reference to be measured by the same analytical technique, preferably, one of a limited group of precise (and expensive, operated by highly qualified personal) techniques with a common measurement standard (e.g., ASTM 1967, ASTM 2926, and ASTM 2927). Classification models, on the other hand, do not require reference samples and, as shown in this work, are more flexible with the techniques and standardization requirement. Thus, laboratories with less than top-of-the-line measurement equipment can still enjoy the benefits of a unified dataset as long as their measurements are self-normalized. While it is undoubtedly better to use proven, standardized, precise analytical methods, real-world constraints require a need for a broader approach that is more inclusive to various analyses and provides additional capabilities than association. On the other hand, classification models such as those developed in this work are only useful in specific scenarios such as hit-and-run accidents where a vehicle leaves glass fragments in the scene of the accident and is not rapidly identified by other means. Moreover, even in such favorable cases, the model can only pinpoint to the car’s manufacturer and not to a specific car.

Still, classification models in general and glass classification models in particular may have multiple forensic usages. In the likely lack of a sufficiently large collection of known samples, glass classification models of cars’ windshields can narrow down the list of potential suspected cars, thereby helping the investigation by excluding false leads. Furthermore, the models presented in this work could be readily extended to cover additional types of glasses (e.g., bottles, windows, and glasses of cultural heritage), and new models could be developed for other types of forensic evidence (arsons, fuels, and gunshot residues).

Yet, the present work still suffers from several caveats, leaves several questions unanswered, and therefore leaves room for improvement. First, as mentioned in previous works,^16^ the examined dataset is relatively small and comprised only 13 vehicles from 10 car manufacturers. Future works should therefore focus on expanding the number of samples and of manufacturers, thus increasing the scope and reliability of the models. Second, considerations pertaining to the supply chain that exists between glass manufacturers and car manufacturers were not taken into account, leaving many questions unanswered including the following: (1) Is there a one-to-one relation between glass manufacturers and car manufacturers? (2) If the same glass manufacturers serve different car manufacturers, is exactly the same glass provided to all? (3) Do car manufacturers perform additional chemical processes on the supplied glass, and if so, what is the nature of these processes? The information obtained by answering these questions could be incorporated into future models to increase their reliability.

Nonetheless, despite these shortcomings, we suggest that the workflow outlined in this work could be beneficially used for the derivation of classification models that may prove useful in multiple forensic domains to the benefit of forensic investigations.

## Conclusions

In this work, we demonstrate that measurements performed on glass fragments using different analytical techniques could be favorably combined into a unified database, provided some threshold conditions are met: (1) Technique-specific measurements are able to afford reasonable classification models. (2) Different techniques can construct classification models using features determined to be important by other techniques. We believe that this work, coupled with previous works, pave the way toward the further exploration of forensic glass evidence by the construction of multi-lab, multi-technique uniform DB. This may form the basis for international-wide collaboration between law enforcement agencies. We emphasize that the conclusions drawn from this work are by no means limited to the glass classification domain and may be similarly applicable to other domains of forensic relevance.

## Data and Software Availability

Python script and data are located on github: https://github.com/omerKaspi/Glass_Classifier.
